# Grade V AAST Intestinal Vascular Injury Secondary to Blunt Abdominal Trauma: A Case Report From a University Hospital in Saltillo, Mexico

**DOI:** 10.7759/cureus.94297

**Published:** 2025-10-10

**Authors:** Alejandra Y Proa-Arriaga, Adrian A Santos-Vega, Oscar A Romero, Gloria Isela Mendoza-Frías, Fernando Martínez-Cuspinera

**Affiliations:** 1 General Surgery, Hospital Universitario de Saltillo, Saltillo, MEX

**Keywords:** aast small bowel, abdominal blunt trauma, acute intestinal ischemia, acute surgical abdomen, bucket-handle mesenteric injury, emergency exploratory laparotomy

## Abstract

A 65-year-old male with no relevant medical history presented to the emergency department with generalized abdominal pain following blunt trauma caused by a bicycle handlebar impact to the mesogastrium 48 hours prior. The patient initially self-medicated with analgesics without improvement. Upon evaluation, he had stable vital signs, epigastric ecchymosis, decreased peristalsis, and signs of generalized peritonism. Laboratory results showed leukocytosis (17.6 x 10^9^/L). Plain abdominal radiographs showed intestinal loops with diameters within normal limits and a few short air-fluid levels in the left hypochondrium. Notably, the distribution of intraluminal gas in that region revealed a “string of pearls” sign, raising suspicion for an obstructive process. An abdominal CT scan revealed inflammatory pelvic changes, mesenteric fat stranding, dilated ileal loops, and free fluid in the pelvic cavity. Exploratory laparotomy showed 150 mL of serosanguineous fluid, a mesenteric border hematoma of the ileum 100 cm from the ileocecal valve, a phlegmon with adhesions, and a 12.5 cm devascularized segment of ileum (bucket handle injury) with necrosis located 120 cm from the ileocecal valve, classified as AAST (American Association for the Surgery of Trauma) Grade V injury and signs of acute appendicitis (erythema and fibrinopurulent coating). Resection with end-to-end anastomosis and appendectomy was performed. The patient tolerated oral intake by postoperative day 2 and was discharged with outpatient follow-up. Histopathology confirmed intestinal necrosis with regional peritonitis and periappendicitis. In patients with blunt abdominal trauma, the most frequently affected organs are the spleen, liver, and small intestine. Injuries to these organs can be missed when the trauma mechanism is underestimated; therefore, frequent physical re-evaluation is essential, as a single examination does not completely exclude the presence of injury.

## Introduction

American Association for the Surgery of Trauma (AAST) Grade V intestinal injuries represent the most severe form of bowel trauma, involving complete transection or devascularization. Although they account for less than 1% of abdominal trauma cases, they carry a high risk of ischemia, sepsis, and mortality exceeding 40%, underscoring the need for early recognition and prompt surgical management. Abdominal blunt trauma (ABT) in adults can result in injuries ranging from solid organ lacerations to more elusive lesions involving the mesentery and small bowel [1,2]. The abdomen ranks as the third most commonly injured anatomical region [3]. The presence of hemodynamic instability or peritonitis in blunt abdominal trauma constitutes an absolute indication for immediate exploratory laparotomy. This approach aims to prevent progression to sepsis and other severe complications, including dehiscence, fistula, or hemorrhage, with a mortality rate reported in the literature of up to 30% [4-6]. While rare, mesenteric vascular injuries carry significant morbidity due to their often-subtle clinical presentation and potential for delayed diagnosis [1,2]. This case report describes a high-grade intestinal vascular injury (AAST Grade V) secondary to blunt abdominal trauma, as well as the surgical approach required for its management.

## Case presentation

A 65-year-old male with no significant past medical history presented to the emergency department 48 hours after falling from a bicycle and striking his mesogastrium against the handlebar. The patient reported progressive abdominal pain, unrelieved by self-administered analgesics, and associated fever. On arrival, he was hemodynamically stable. Physical examination revealed epigastric ecchymosis, decreased peristalsis, generalized abdominal dullness, and positive McBurney and Rovsing signs.

Laboratory tests revealed leukocytosis, neutrophilia and hyperglycemia (Table 1).

**Table 1:** Laboratory results Laboratory results of the patient compared with corresponding reference ranges. Values outside the reference range are highlighted to indicate potential clinical significance.

Parameter	Patient Value	Reference Range
Hemoglobin (g/dL)	15	12–16 g/dL
Platelets (x10^3^/µL)	242	150–400 x 10^3^/µL
Leukocytes (x10^3^/µL)	17.6	4.5–11 x 10^3^/µL
Neutrophils (%)	87.2	40–70%
Glucose (mg/dL)	130	70–110 mg/dL
Blood Urea Nitrogen (BUN) (mg/dL)	16.5	7–20 mg/dL
Creatinine (mg/dL)	1.1	0.6–1.2 mg/dL
Total Protein (g/dL)	7	6–8.3 g/dL
Albumin (g/dL)	4	3.5–5.0 g/dL
Amylase (U/L)	46	30–110 U/L
Lipase (U/L)	11	0–60 U/L
Sodium (mmol/L)	136	135–145 mmol/L
Chloride (mmol/L)	99	98–106 mmol/L
Potassium (mmol/L)	4.7	3.5–5.1 mmol/L
Prothrombin Time (s)	11.2	11–13.5 s
International Normalized Ratio (INR)	1	0.8–1.2
Partial Thromboplastin Time (s)	25.3	25–35 s

**Figure 1:**
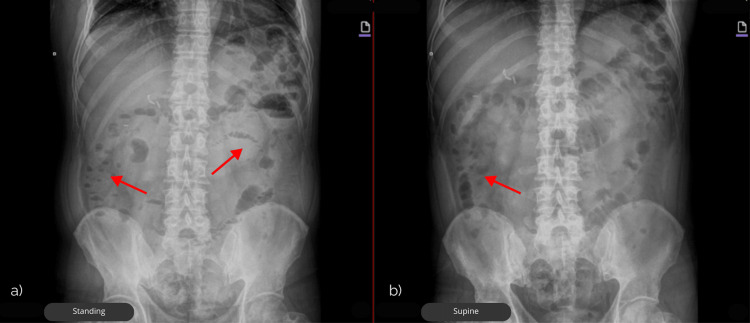
Plain abdominal X-rays in two projections showing short air–fluid levels and the “string of pearls” sign, as indicated by the red arrows in (a) standing and (b) supine positions, suggestive of intestinal obstruction.

Plain abdominal radiographs in two positions showed intestinal loops with diameters within normal limits and a few short air-fluid levels in the left hypochondrium. Notably, the distribution of intraluminal gas in that region revealed a “string of pearls” sign, raising suspicion for an obstructive process (Figure 1).

**Figure 2:**
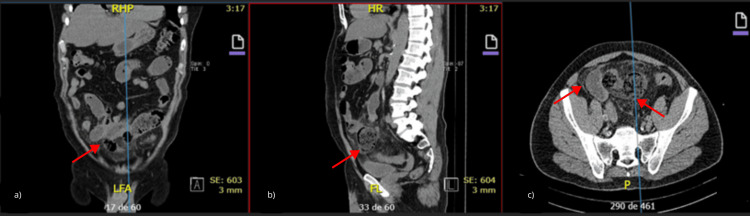
Abdominal CT demonstrating transition point and proximal dilatation Abdominal computed tomography (CT) in (a) coronal, (b) sagittal, and (c) axial sections demonstrating a pelvic inflammatory process with mesenteric fat stranding, a dilated ileal loop with fecaloid content at the transition point, dilated proximal jejunal loops with fluid, and free fluid in the pelvis (red arrows).

Abdominal non-contrast computed tomography (CT) imaging showed pelvic inflammation, increased mesenteric fat density, dilated ileal loops with fecaloid content, proximal jejunal dilation with intraluminal fluid, and free fluid in the pelvic cavity (Figure 2).

After a non-contrast CT showed a transition point with proximal dilatation, mesenteric fat stranding, and free fluid, the patient’s persistent acute surgical abdomen prompted an exploratory laparotomy.

**Figure 3:**
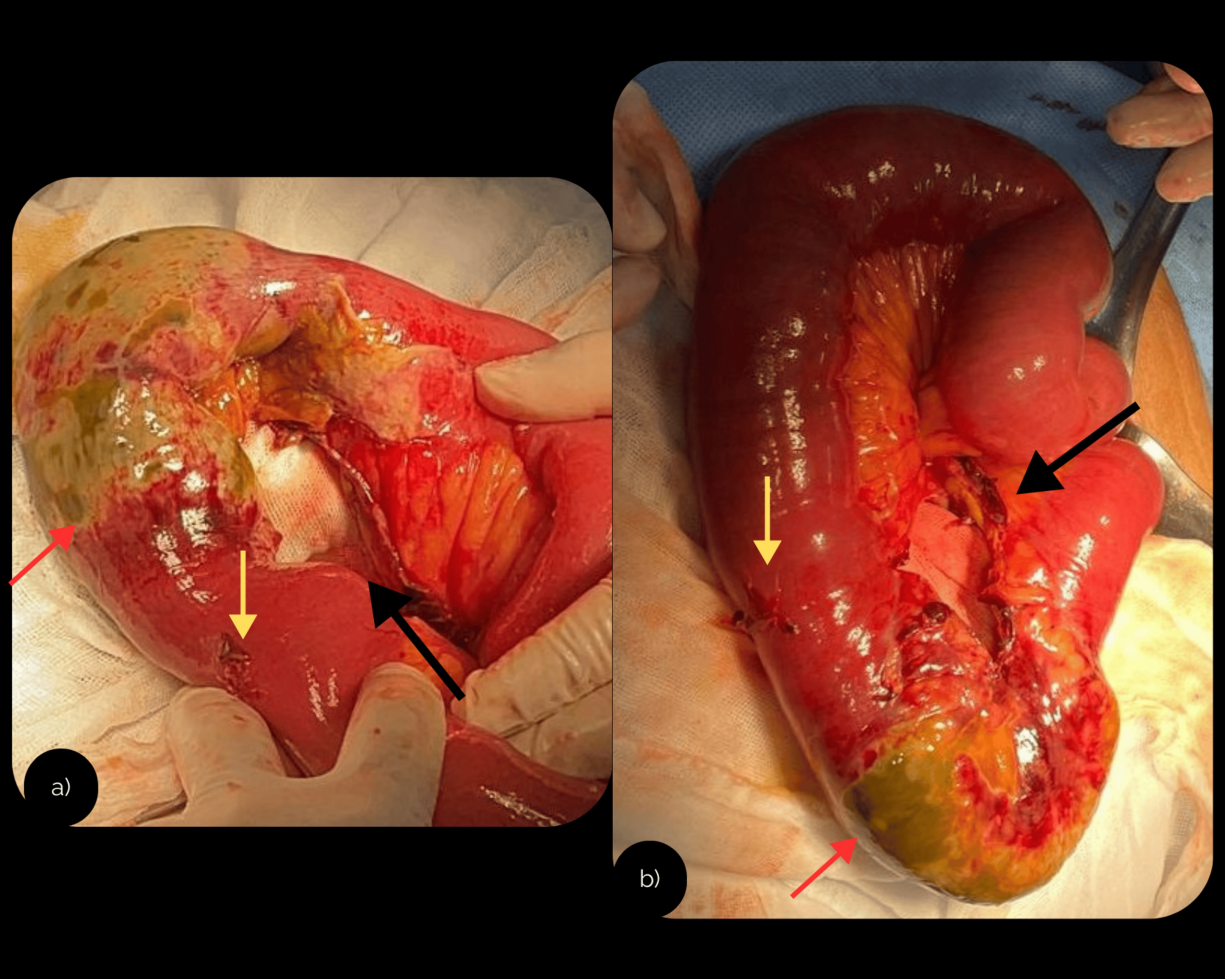
Exploratory laparotomy: intraoperative images. Intraoperative photographs taken during exploratory laparotomy showing different angles of the same lesion. (a, b) Bucket-handle injury of the ileum (black arrow), associated with loss of viability of an ileal segment (red arrow) and serosal disruption of the ileum (yellow arrow).

Intraoperative findings included approximately 150 mL of serosanguineous fluid, a 1 cm hematoma on the mesenteric border of the ileum 100 cm proximal to the ileocecal valve, and an inflammatory phlegmon with dense adhesions between ileal loops, the abdominal wall, and the omentum. A 12.5 cm segment of devascularized ileum with necrosis (bucket-handle sign) was identified 120 cm from the ileocecal valve (Figure 3) consistent with AAST Grade V injury, and involving ischemic changes to the distal third of the cecal appendix (edematous, erythematous, and covered with fibrinous coating).

**Figure 4:**
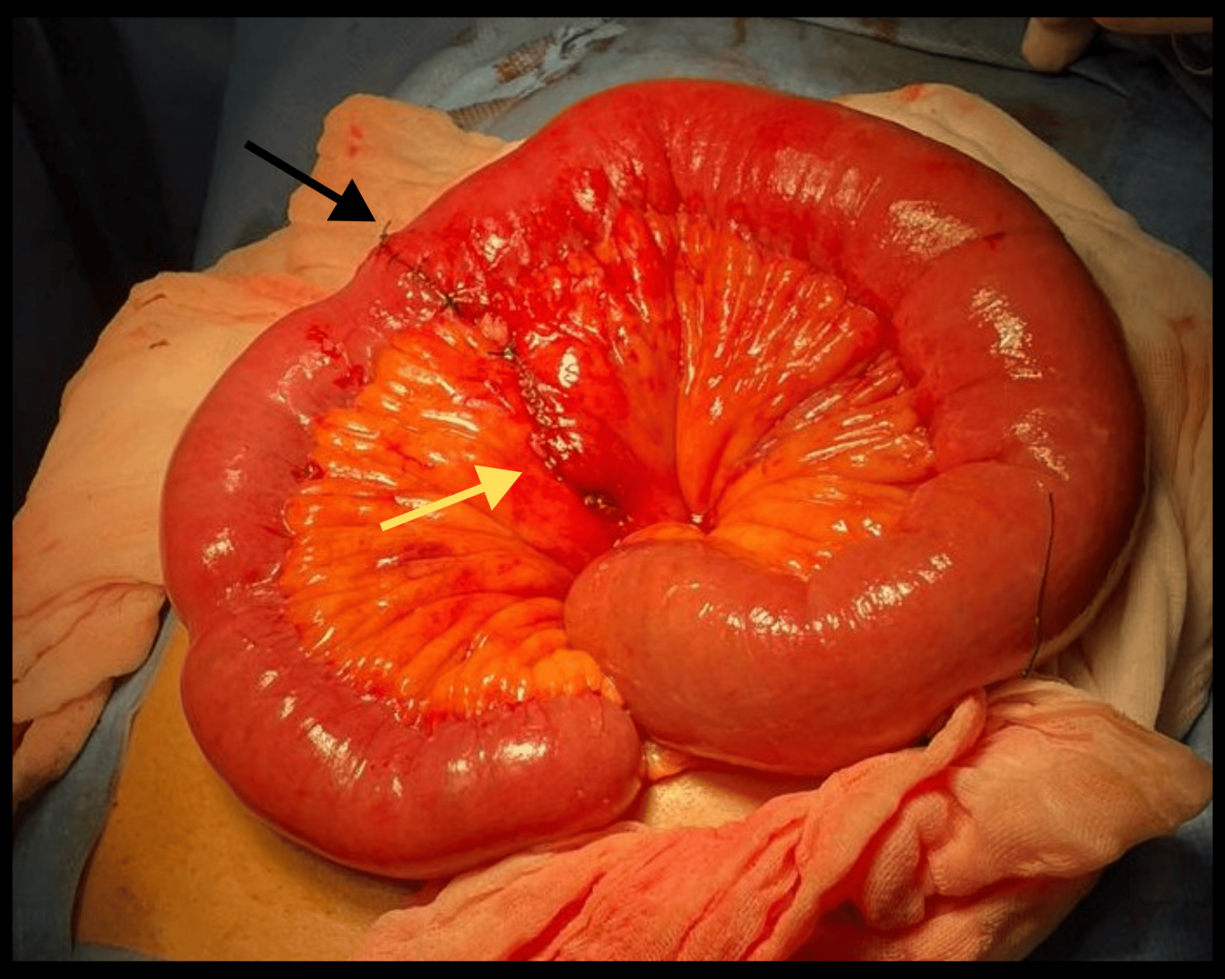
Intraoperative outcome of the procedure: anastomosis with closure of the mesenteric defect. A first-layer anastomosis (black arrow) was performed using Connell-Mayo sutures with 2-0 Vicryl, reinforced with a second seromuscular layer using Lembert sutures with 2-0 silk. The mesenteric defect (yellow arrow) was closed with a continuous 2-0 silk suture.

Resection of the devascularized segment (approximately 15 cm) was performed, followed by a single-layer anastomosis using Connell-Mayo sutures with 2-0 Vicryl, and a second seromuscular layer with Lembert sutures using 2-0 silk (Figure 4).

The patient had an uncomplicated postoperative course, tolerated oral feeding by postoperative day 2, and was discharged with scheduled outpatient follow-up. Final histopathological analysis confirmed necrotic ileum with regional peritonitis and periappendicitis.

## Discussion

Intestinal vascular lesions following blunt trauma pose diagnostic challenges due to their often-subtle onset [3]. This case illustrates the importance of maintaining a high index of suspicion in patients with blunt abdominal trauma and highlights the role of early imaging and surgical intervention in preventing complications.

Blunt abdominal trauma with intestinal or mesenteric injury is uncommon but potentially life-threatening. Duodenal injury is often diagnosed late due to the retroperitoneal location of the duodenum and is associated with high rates of complications and mortality [2]. The main mechanism of injury is crushing (blunt trauma), caused, for example, by the handlebar of a bicycle or the steering wheel of a car [7]. A less common cause is deceleration, which more frequently results in damage to the third and fourth portions of the duodenum [7].

In hemodynamically stable patients with suspected intra-abdominal injury, CT serves as an adjunct to clinical assessment [8]. In this case, a non-contrast CT showed indirect signs (transition point, mesenteric fat stranding, and free pelvic fluid) that supported - rather than determined - the decision for exploratory laparotomy prompted by persistent peritoneal signs.

A 2019 meta-analysis evaluated over 1,000 patients with blunt abdominal trauma and found conservative management successful in 60%, with 40% requiring surgical intervention [3], and highlighted the importance of early diagnosis and multidisciplinary collaboration in optimizing outcomes.

The classification of small bowel injuries proposed by Moore et al. provides a standardized grading system to assess trauma severity, ranging from Grade I (hematoma without devascularization) to Grade V (bowel transection with segmental tissue loss or complete devascularization) [9]. In this system, an additional category includes isolated vascular injuries, which are also classified as Grade V. This grading scale is particularly useful for correlating radiological findings with intraoperative damage and for guiding surgical decision-making [9]. In cases involving multiple intestinal injuries, the authors recommend increasing the grade by one level, up to a maximum of Grade III, as shown in Table 2.

**Table 2:** Table representing the classification of small bowel injuries according to the AAST (American Association for the Surgery of Trauma).

Grade	Type of injury	Description of injury
I	Hematoma	Contusion or hematoma without devascularization
I	Laceration	Partial thickness, no perforation
II	Laceration	Laceration <50% of circumference
III	Laceration	Laceration ≥50% of circumference without transection
IV	Laceration	Transection of the small bowel
V	Laceration	Transection of the small bowel with segmental tissue loss
V	Vascular	Devascularized segment

This case underscores a low threshold for exploration when indirect CT signs persist alongside peritoneal findings, even with non-contrast CT. Once segmental devascularization is confirmed, limited resection with primary anastomosis is appropriate and was associated with an uncomplicated recovery in our patient [1,4,6]. An appendectomy was performed due to an inflamed-appearing appendix; final histopathology showed periappendicitis (serosal inflammation), consistent with a reactive/incidental finding.

This case contributes to the limited literature on high-grade AAST intestinal vascular injuries and emphasizes the need for vigilance in evaluating patients with delayed presentation after blunt trauma.

## Conclusions

High-grade mesenteric and small bowel vascular injuries, although rare, must be considered in blunt abdominal trauma with persistent or worsening symptoms. They can present late and with subtle imaging findings. In our patient, non-contrast CT demonstrated indirect signs (transition point, mesenteric fat stranding, free pelvic fluid) that supported - but did not replace - clinical judgment in the context of a surgical abdomen; early laparotomy with limited resection led to an uncomplicated recovery. CT should be used as an adjunct to identify additional or missed injuries and to aid surgical planning, while maintaining a low threshold for operative exploration when peritoneal signs persist despite equivocal imaging.
